# Development of a Prototype Miniature Silicon Microgyroscope

**DOI:** 10.3390/s90604586

**Published:** 2009-06-11

**Authors:** Dunzhu Xia, Shuling Chen, Shourong Wang

**Affiliations:** School of Instrument Science and Engineering, Southeast University, Nanjing City, Jiangsu Province, 210096, China; E-Mails: chenshuling318@126.com (S.C.); srwang@seu.edu.cn (S.W.)

**Keywords:** silicon microgyroscope (SMG), self-oscillating, dual-channel closed-loop, scale factor, zero bias stability, temperature compensation, miniature prototype

## Abstract

A miniature vacuum-packaged silicon microgyroscope (SMG) with symmetrical and decoupled structure was designed to prevent unintended coupling between drive and sense modes. To ensure high resonant stability and strong disturbance resisting capacity, a self-oscillating closed-loop circuit including an automatic gain control (AGC) loop based on electrostatic force feedback is adopted in drive mode, while, dual-channel decomposition and reconstruction closed loops are applied in sense mode. Moreover, the temperature effect on its zero bias was characterized experimentally and a practical compensation method is given. The testing results demonstrate that the useful signal and quadrature signal will not interact with each other because their phases are decoupled. Under a scale factor condition of 9.6 mV/^°^/s, in full measurement range of ± 300 deg/s, the zero bias stability reaches 15^°^/h with worse-case nonlinearity of 400 ppm, and the temperature variation trend of the SMG bias is thus largely eliminated, so that the maximum bias value is reduced to one tenth of the original after compensation from -40 ^°^C to 80 ^°^C.

## 1. Introduction

The silicon microgyroscope (SMG) is an important kind of sensor found in inertial instruments that are widely used in aerospace measurement, balance control, and vehicle navigation systems. One of the major features for successful commercialization of microgyroscopes is their vacuum packaging under reduced pressure levels, which is essential in determining reliability, working performance, required drive voltage and the total cost of the sensor module. The performance of SMGs under vacuum has been studied intensively, and some gyroscopes are found to have a better performance in a vacuum chamber [1,2]. Some mature vacuum packaging technologies such as silicon-silicon bonding and silicon-glass wafer bonding are successfully used to package microgyroscopes [3]. In this paper, a special and timely effective packaging approach using laser welding has been realized in order to guarantee the gyroscope will function under high mechanical sensitivity conditions i.e., with a high quality factor (Q factor), in a vacuum environment and for a long time.

Apart from the preparation of a suitable vacuum working environment, the meticulous design of structure and circuitry become the two main key technologies. Considering that the previous literature primarily used varying-distance detection which could cause unexpected nonlinearity errors, a fully decoupled structure with varying-area style is proposed. In most of the literature drive mode circuitry designs of AGC and PLL (Phase Lock Loop) modules have been independently or simultaneously used to attain a certain performance [4-6]. On this basis, a simplified drive circuit with a combination of AGC and modulation technologies was investigated to achieve a high SNR (Signal Noise Ratio) and good performance. As is known, open-loop detection is favorable for design simplification and to accomplish the integration and miniaturization of the measurement-control circuit, which can thus basically meet the specification requirements of low-precision SMGs, and has been successfully applied in many reports [2,3]. Unfortunately, the linearity of open-loop detection tends to be affected by the non-linear characteristics of detection displacement and its corresponding capacitance, which leads to a limited measurable angular range. Especially, the gyroscope's use is complicated, usually affected by many kinds of disturbances such as outer interferences, over shocks, vibrations, temperature and so on. Though some gyroscopes in the literature [7,8] use single channel closed-loops or double-channel closed-loops without modulation of high frequency carrier signal, it is very difficult for a separate charge amplifier to measure a minor varying signal due to some large existing unquenchable parasite signals.

Some ASIC gyroscopes using sigma-delta digital closed-loop has been realized in the lab [9-11], but this architecture is more complex to realize in separate circuitry. In [12], though digital control loops are successfully perfected for basic performance, two carrier signals (600 kHz and 400 kHz) are required for modulation in the drive and sense modes, respectively, which will increase the circuit complexity. Only a PI (Proportion-integral) controller is designed to control the structure rather than a PID (Proportion-integral-derivative) module, which may result in incomplete control, especially while the microgyroscope works in a complex dynamic environment. Moreover, the adjustable phase reference for modulation is very simple such as 0^°^ and 90^°^. As for the actual gyroscope, the phase shift is actually very difficult to anticipate because the signal phase will be effected by both circuitry and two unmatched modes of the structure, which needs an adjustable phase shifter.

In this design, only one carrier signal (1 MHz) is input to the proof mass port for simultaneous modulation of both drive and sense modes. On the other hand, the open-loop system bandwidth is approximatively decided by the difference between resonant frequencies in two modes, and it is quite difficult to adjust them once the structure is produced. In this paper, an effective resolution is found to regulate the bandwidth using PID compensator module in the closed-loop system.

Above all, in order to develop a miniature silicon microgyroscope, a newly improved type of structure with vacuum packaging was investigated first. In drive mode, a self-oscillating drive circuit will be designed to ensure a constant motion, and in sense mode, a dual-channel closed-loop detection method using carrier signal modulation and demodulation in pure separate analogy components is presented and implemented successfully. Meanwhile, since there always exists zero bias drift with temperature [13,14], a practical digital compensation system using microprocessor should be designed to attenuate the temperature influence.

## 2. Design of the SMG Structure

In terms of structure design, decoupling, sensitivity, and linearity there are three main important rules. Most conventional decoupled microgyroscopes have two or more degrees-of-freedom (DOF) to ensure their decoupling but ignoring their linearity. Though the combination of linear-linear structure and rotary-rotary structure achieves a good decoupling performance [15], the nonlinearity becomes prominent because of its arched capacitor plates. Another problem is that most decoupled gyroscopes adopt varying-distance detection in sense mode, despite having a good symmetrical style, which will also cause a serious nonlinearity while working at a comparatively large angular rate.

**Figure 1.**
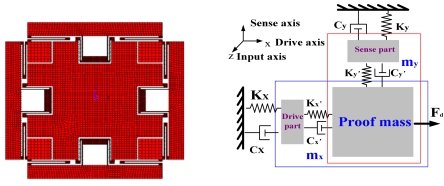
The structure and model of SMG.

Based on the structure in [16], which cannot be a completely symmetric structure because of its varying-distance style in sense modes, a newly improved type of structure is designed. What is special is that drive mode and sense mode are completely symmetric because they are both varying-area style. A simplified model of the SMG is shown in Figure 1.

In this design, a varying-area detection method is applied in sense mode, which ensures a better linearity than the former mentioned gyroscopes. In addition, this gyroscope, with slide-film damping, has a quality factor nearly 10 times higher than squeeze-film damping at the same ambient pressure, in spite of about 1/10 conversion coefficient from displacement to capacitor, so the general sensitivity to Coriolis force is at the same level for both varying-distance and varying-area styles. In order to improve its sensitivity, multi rows of interdigital comb-finger capacitors are arranged orderly to amplify the displacement-capacitive-ratio conversion. Folded beams are anchored toward the inner direction of the proof mass and have an excellent symmetry in both drive and sense mode. In the actual structure, an added stop block is designed in the center of the proof mass to implement the over shock preventing function.

The symmetrical and decoupled microgyroscope designed in this paper is essentially a linear vibratory gyroscope to detect rotation angular rate by the oscillating components. Its simple model can be described as a total four DOFS system including proof mass, drive component, sense component, and damping elements. Specifically, the drive component has one DOF in the drive direction, the sense component has one DOF in the sense direction, and the common proof mass has two DOFs in two directions. In this model, m_x_ is the general mass with the sum of drive part and proof mass in drive direction, K_x_ and C_x_ are respectively the general stiffness coefficient and damping coefficient in drive direction, K_x'_ and C_x'_ are the inner stiffness coefficient and damping coefficient between drive part and proof mass respectively. Similarly, m_y_ is the general mass with the sum of sense part and proof mass in sense direction, K_y_ and C_y_ are respectively the general stiffness coefficient and damping coefficient in sense direction, K_y'_ and C_y'_ are the inner stiff coefficient and damping coefficient between sense part and proof mass respectively. F_d_ is the electrostatic force to drive the structure along x-axis.

According to the design rule of all the folded beam stiffness (the stiffness in the required direction is greater than that in its orthogonal direction), it is obvious that both the drive and sense components primarily vibrate in its corresponding DOF. In other words, the sense component almost vibrates in sense direction even if the drive component sufficiently vibrates in drive direction. So the coupling between the two modes is enormously reduced. Its working mechanism includes that:

1. The drive component is driven as steadily as possible to ensure the drive frequency and vibrating amplitude are both stable and constant.
2. Due to the Coriolis effect, the Coroilis force along the sense direction is generated when a rotation happens in the perpendicular direction.
3. Because the vibratory amplitude in sense mode is in direct proportion to the outer angular rate, the gyroscope can detect the corresponding angular rate through detecting the vibratory amplitude.

Additionally, use of a varying-area style in the sense part and in the drive part can ensure high sensitivity and linearity in the detection of the Coriolis signal. For simplicity, ignoring the inner force between proof mass and other two parts (drive part and sense part), the symmetrical and decoupled microgyro motion equations are generalized [17] by:

(1) $$m_{x}\ddot{x}(\omega t)+C_{x}\dot{x}(\omega t)+K_{x}x(\omega t)=m_{x}\left[\ddot{x}(\omega t)+\frac{\omega_{nx}}{Q_{x}}\dot{x}(\omega t)+\omega_{nx}^{2}x(\omega t)\right]=F_{d}+k_{\text{xy}}y(\omega t)$$

(2) $$m_{y}\ddot{y}(\omega t)+C_{y}\dot{y}(\omega t)+K_{y}y(\omega t)=m_{y}\left[\ddot{y}(\omega t)+\frac{\omega_{ny}}{Q_{y}}\dot{y}(\omega t)+\omega_{ny}^{2}y(\omega t)\right]=-2m_{y}\Omega_{z}\dot{x}(\omega t)+k_{yx}x(\omega t)+ɛx(\omega t+\varphi )$$

where *x* and *y* are displacements in drive and sense modes, *ω_nx_* and *ω_ny_* are resonant frequencies in two modes, *Q_x_* and *Q_y_* are quality factors in two modes, *F_d_* is the electrostatic driving force in drive mode, Ω*_z_* is the input angular rate in *z* direction, -2*m_y_*Ω*_z_ẋ* is the Coriolis force componentô*k_xy_y* and *k_yx_x* denote the forces generating orthogonal coupling signal in two modes, *εx*(*ωt+φ)* is the force generating offset error signal, which will cause offset output without the rotation of gyroscope, *ε* is a proportional coefficient between the force and the displacement, *φ* is a actual phase shift which will decide the phase relationship among the offset error signal, Coriolis signal and quadrature signal. In [18], the researchers have intensively studied its mathematic model and recognized its sources, which are different from the Coriolis and quadrature signals. Actually, their phase relationship is such that the Coriolis and quadrature signals are orthogonal, the offset error signal is not in phase with them when the modes are unmatched, but the offset error signal is in same phase as the Coriolis signal if the modes are matched.

**Figure 2.**
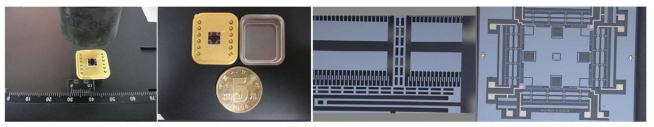
The package and SEM photos of a SMG structure.

Structure design and fabrication imperfections are always major factors resulting in quadrature and offset errors [18,19]. In theory, these imperfections are reflected in asymmetry and anisoelasticity of the structure, and can be captured as misalignment of principal axes of elasticity from intended axes of symmetry of the structure. There would potentially be a solution to reduce stiffness and displacement coupling coefficients mentioned previously by using this special symmetrical and decoupled style. Figure 2 shows the package and SEM photos of a SMG structure.

## 3. Design Schemes of the SMG in Drive and Sense Modes

### 3.1. Self-Oscillating Scheme in Drive Mode

Currently most SMGs utilize capacitive electrostatic excitation, which can guarantee that the driving frequency ω_d_ approaches the natural frequency ω_nx_ as close as possible in drive mode, and the amplitude is as stable as possible. To satisfy these requirements, closed-loop control must be achieved in the actuation of the SMG. In order to achieve the self-oscillating startup of an oscillator, the closed-loop control must meet two requirements, i.e., the phase angle of the whole loop θ = 2nπ (n is an integer) and the gain of the whole loop A > 1 [20]. The closed-loop actuation of an SMG commonly adopts AGC, which implements the closed-loop driving with a non-linearity dynamics characteristic, reducing the closed-loop gains of the entire loop to 1 [21]. In most of previously mentioned literature [4-6], AGC and PLL modules have been used successfully. However, it is difficult to simplify the electronics when both these modules are used. On the other hand, tracking accuracy is also affected by the presence of Brownian noise and capacitive position sensing noise [22]. Due to the complicated Micro-Electro-Mechanical interface between all kinds of plate electrodes of the gyroscope, the accurate detection of the driving motion using a large bias voltage Vp (even 40 V) is difficult to carry out because of a variety of unknown parasitic capacitances and low SNR in actual separate components, which will simultaneously cause a large power consumption.

Considering the above reasons, a high SNR detection of driving motion using high-frequency (1 MHz) carrier-signal modulation and demodulation, and a wide flexible phase adjustment by a precision phase shifter are both adopted to improve the scheme of self-oscillation-driven of the SMG. The AGC goal is achieved through adding an automatic varying DC (direct current) voltage V_dc_ with the AC (Alternating current) voltage signal to produce a servo driving force, which can make the drive mode vibration amplitude constant.

The scheme succeeds in decoupling the phase angle and gain of the self-oscillation-driven, where the phase angle and the gain can be irrelevant and adjusted separately, which thereby increasing the stability of drive frequency and the amplitude. From the analysis above, the role of closed-loop control of driving circuit is to reduce the resonant frequency deviation, i.e., |Δω/ω_d_| as small as possible and increase the stability of the driving frequency and amplitude.

**Figure 3.**
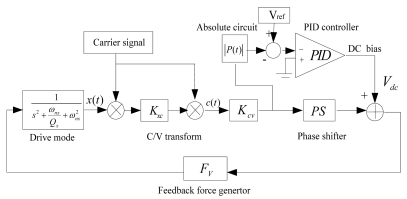
Drive mode circuitry method.

As shown in Figure 3, a whole scheme of driving circuit method is realized by orderly composition of the SMG's transfer function in drive mode, modulation module, K_xc_ module (the transfer coefficient from combo finger displace to its equivalent capacitance), demodulation module, K_cv_ module (the transfer coefficient from capacitor to voltage), AGC loop, phase shifter module, and force feedback generator module F_v_. Especially, a high frequency precision carrier (1 MHz) is provided for modulation and demodulation. In the AGC loop, a PID controller module can ensure that the oscillating amplitude of the gyroscope in drive mode is equal to the set value by adjusting V_ref_, and the absolute value circuit can complete the rectification of the demodulated AC signal.

**Figure 4.**
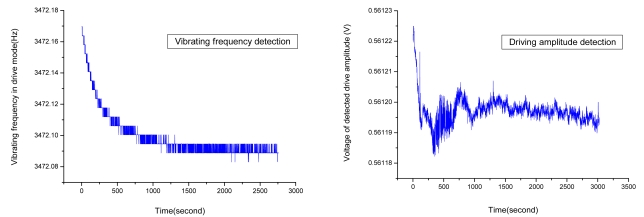
Vibrating frequency and amplitude in drive modes.

One hour detection results of vibrating frequency and amplitude in drive mode are shown in Figure 4. Since the power startup, the mean value of the vibrating frequency in drive mode is near 3,472.1 Hz, and its variance is 0.01538 Hz. Similarly, the mean detection voltage value of vibrating amplitude in drive mode is near 0.5612 V, and its variance is 4.49182 × 10^-6^ V, so the stability of both vibrating frequency and amplitude in drive mode have achieved a high precision (the relative error is lower than 1 × 10^-5^).

### 3.2. Dual-Channel Closed-Loop Detection of SMG

Due to a variety of advantages over open-loop such as reduced noise and better stability, all kinds of digital or analog closed-loops are adopted in the gyroscope detection circuits in some literature [6-12,23]. In digital closed-loop [9-11], a sigma-delta interface can be functionally implemented to detect the minor motion signal, but the switch noise from sequence control clock probably becomes a noise source if used in the separate circuits here without careful treatment. In addition, a signal-channel closed-loop control used in sense mode cannot completely counteract the useful signal, the quadrature signal and offset error, because the force feedback is phase insensitive before performed synchronous demodulation to Coriolis plus offset and quadrature plus offset components.

**Figure 5.**
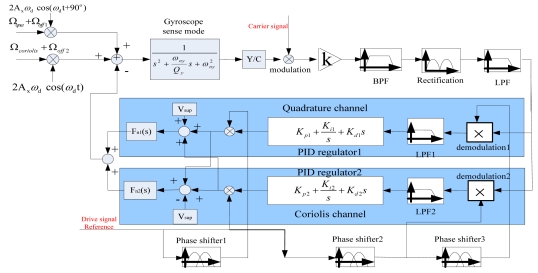
Sense mode circuitry method.

In [7] even three loops in analog closed-loop were applied without consideration of the concrete phase relationship among the above signals, Especially, when there always exits mismatch (resonant frequency difference) in two modes of gyroscope, the phase relationship of them is complex to need modification and demodulation to reach a better sensitivity. In all designs mentioned above, the motion signal detection is difficult to reach high SNR because of all kinds of the parasite signals, which mainly arise from the complex Micro-Electro-Mechanical interface and appear in low frequency band near resonant frequency. In [24,25] two-channel decomposition and reconstruction closed loops are applied, the testing results demonstrate that the useful and quardrature signal will not interact because of their phase decoupling. However, the controlled gyroscope named szg3 is varying-distance style, and here the newly designed varying-area style gyroscope named E17 is investigated with the similar control strategy and normalized control parameters. Though the simulated control model is the same as before, due to using improved capacitance detection converter and other high performance circuitry, the actual testing results in this work will be verified to show the obvious improvement for this overall system. In this case, the whole control method will be further analyzed in detail. Based on our previous work, an improved dual-channel closed-loop detection block diagram of this proposed kind of SMG mentioned above is given in Figure 5, which will take full advantage of modulation and demodulation method of high frequency carrier, three adjustable precision phase shifters, and optimal PID compensators.

**Figure 6.**
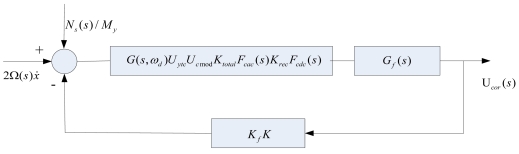
The simplified block diagram of coriolis signal closed-loop detection.

The output signals of the SMG are appropriately complicated; they principally contain two parts which are orthogonal to each other: quadrature part and Coriolis part. The quadrature part contains the quadrature coupling signal and one part of the offset signal. The Coriolis part contains the Coriolis signal and the other part of the offset signal. For this gyroscope with a better decoupled structure, the offset error signal is relatively so weaker than the Coriolis signal that it is easy to cut off the interfering signal from the Coriolis signal. According to the theory of signal processing, the optimal method of separating the interfering signal from the Coriolis signal is the precision phase modulation and demodulation. In Figure 5, the input signals in the Coriolis and quadrature channels are expressed respectively as:

(3) $$S_{\text{coriolis}}(t)=2A_{x}\omega_{d}\cos(\omega_{d}t)•(\Omega_{\text{coriolis}}+\Omega_{\text{off}2})$$

(4) $$S_{\text{qua}}(t)=2A_{x}\omega_{d}\cos(\omega_{d}t+90^{o})•(\Omega_{\text{qua}}+\Omega_{\text{off}1})$$

where A_x_ is the oscillation amplitude in drive mode, ω_d_ is driving frequency same as its natural frequency ω_nx_ according to the designed structure parameters, Ω_coriolis_ and Ω_qua_ are the input angular rates of the Coriolis signal and equivalent quadrature signal, respectively, and they are 90 degree out-of-phase. Ω_off1_ and Ω_off2_ are two decomposition components of offset error in phase of Ω_coriolis_ and Ω_qua_. According to superposition principle in control theory, both of them can be analyzed separately. The common forward part, including transfer function of gyroscope in sense mode, Y/C module (the conversion coefficient from displacement in sense direction to variable capacitor), K (the total gain of amplifiers), BPF (band-pass filter), Rectification module (for first demodulation of both two signals), and LPF (low-pass filter), can pick off these mixed signals. There are two channels including quadrature channel and Coriolis channel, which are similarly composed of the secondary demodulation module, LPF, PID compensator, and electrostatic force feedback generator F_n_(s) by adjusting the preloaded voltage V_sup_, so the whole block diagram constitutes the dual-loop detection of the gyroscope in sense mode. According to the block diagram of dual-channel closed-loop control, Figure 6 illustrates the simplified control scheme of closed-loop detection only in the Coriolis signal channel because these two channels have the same systemic framework. Through this control strategy, the final effect is that the quadrature part is almost completely suppressed and the output signal can automatically follow the input angular signal Ω_coriolis_.

As shown in Figure 6, 2Ω(*s*)*ẋ* is the Coriolis acceleration, N_s_(s)/M_y_ is equivalent to the input angular rate noise. K_f_K is the actuator and adjustable feedback gain. G(s, ω_d_)K_ytc_K_cmod_K_total_F_cac_(s) K_rec_ F_cdc_ (s) is the total forward path transfer function, where G(s, ω_d_) is the transfer function of gyroscope in sense mode, K_ytc_ and K_cmod_ are gain coefficients from Y/C module and carrier signal modulation respectively, K_total_ and K_rec_ are gain coefficients from the total amplifiers and Rectification module respectively. F_cac_(s) and F_cdc_ (s) are transfer functions of BPF and LPF, respectively. G_f_(s) is PID compensator. U_cor_(s) is the output signal.

In fact, the principle of the dual-channel closed-loop detection achieved by the quadrature channel is as same as which achieved by the Coriolis channel. Both signals processed have the same frequency that equal the natural frequency in the SMG's drive mode, the difference between both of them are phase angle and the varying trend, so the closed-loop detection with these two channels, essentially, is an AC feedback servo system which is rapidly controlled to keep the balance of AC force. It means that there would be almost a null displacement of the proof mass in the sense direction. Considering a special AC force feedback, these two channels need both modulation and demodulation, which can achieve a quick closed-loop servo control. These two orthogonal channels are decoupled and do not interfere with each other, so the closed-loop control performance of the two channels can be analyzed separately. For the useful signal channel, ie. the Coriolis channel, the closed-loop transfer function of the closed-loop system is calculated as follows:

(5) $$\text{Let}\;G_{0}(s)=G(s,\omega_{d})K_{\text{ytc}}K_{c\mathrm{mod}}K_{\text{total}}F_{\text{cac}}(s)K_{\text{rec}}F_{\text{cdc}}(s)\;\text{and}\;G(s,\omega_{d})=1/s^{2}+\frac{\omega_{ny}}{Q_{y}}s+\omega_{ny}^{2}$$

then:

(6) $$\begin{array}{ll} G_{c}(s) & =\frac{G_{o}(s)G_{f}(s)}{1+G_{o}(s)K_{f}G_{f}(s)}\\ & =\frac{\frac{K_{\text{ytc}}K_{c\mathrm{mod}}K_{\text{total}}F_{\text{cac}}(s)K_{\text{rec}}F_{\text{cdc}}(s)}{s^{2}+\frac{\omega_{ny}}{Q_{y}}s+\omega_{ny}^{2}}(K_{p}+\frac{K_{p}}{T_{i}}•\frac{1}{s}+K_{p}\tau s)}{1+\frac{K_{\text{ytc}}K_{c\mathrm{mod}}K_{\text{total}}F_{\text{cac}}(s)K_{\text{rec}}F_{\text{cdc}}(s)}{s^{2}+\frac{\omega_{ny}}{Q_{y}}s+\omega_{ny}^{2}}K_{f}(K_{p}+\frac{K_{p}}{T_{i}}•\frac{1}{s}+K_{p}\tau s)} \end{array}$$

where the PID compensator is:

(7) $$G_{f}(s)=K_{p}+\frac{K_{p}}{T_{i}}•\frac{1}{s}+K_{p}\tau s$$

**Figure 7.**
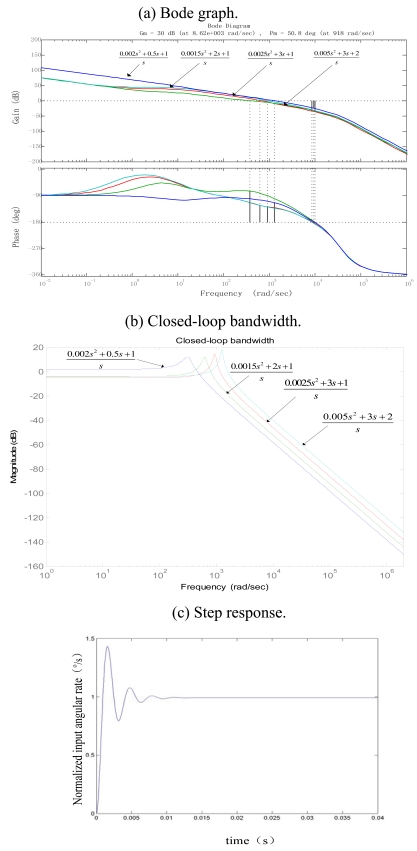
The simulated performances of the whole closed-loop system.

Obviously, the closed-loop transfer function is a high-order system. It can be computed through Matlab tools. The effect of the PID compensator's parameters on the system bandwidth is shown in Table 1, and Figure 7 shows the result of simulation.

As shown in Table 1, changing the transfer function of the compensator module (just changing the PID parameters: the proportional coefficient *K_p_*, time integral constant *T_i_*, time differential constant *τ*) can influence the closed-loop system bandwidth of gyroscope. Meanwhile, Figures 7(a), (b) illustrate the changes of closed-loop system bandwidth with different compensator models, and the system Bode graph shows the changes of gain margin and phase margin. Therefore the chosen parameters of the designed compensator should not only meet the system bandwidth requirements, but also guarantee the stability of the system. From the above analysis, there exists a complex nonlinear relationship between the bandwidth and compensator parameters. However the tradeoff among these parameters can be found to reach a certain bandwidth together with a better stability by Matlab simulation tools. In this case, when *K_p_* approaches near 2, time integral constant *T_i_* approaches near 2s, and time differential constant *τ* approaches near 0.0075 s, the closed-loop system shown in the Figure 7(b) displays its stability and reliability with the phase margin greater than 45 degree ,the amplitude margin more than 30 dB, and the bandwidth approaching 100 Hz. Though the bandwidth can be further increased by two later compensator parameters, the corresponding phase and amplitude margins tend to drop almost near to 45° and 30 dB, which demonstrates that the instability of closed-loop. The step response in Figure 7(c) displays a small overshoots less than 30 percent and quick response time near 5 ms when input angular velocity of rotation is 1^°^/s.

## 4. Digital Temperature Compensation

The proposed silicon micro-machined gyroscope is made of silicon, which would be influenced by temperature variation, resulting in the distortion of the structure, the change of the Young's modulus, the resonant frequency, and the Q factor etc. The effects of the environmental temperature on the performance is of great interest, since they can be an important source of error in gyroscopes. Essentially, temperature variation will affect the Young's modulus of the material and result in deformation of the structure, deviations in resonant frequencies and change of Q factor. Therefore the output of the system has a complicated changing process, with the result that the bias will drift greatly owing to the temperature variation. The temperature dependent characteristics of the gyroscope were investigated without compensation resolution in [13,14]. In order to eliminate the impact from the temperature variation, a temperature compensation system is designed which can calculate the compensation values at each temperature point ranging from -40 °C to 80 °C in terms of the compensation model previously set up by experiments .

### 4.1. The Mechanism of the Temperature Effect

Because *L* is the parameter of both Young's modulus and the spring stiffness *K*, all of them would be changed with temperature variation. Suppose that the coefficient of thermal expansion of silicon is α, and the initial length of silicon structure is *l_0_*. The temperature change from initial *T_0_* to *T* will result in the change of the three physical quantities as follows:

(8) $$L=l_{0}\left[1+\alpha (T-T_{0})\right],\;E\left(T\right)=E_{0}-E_{0}k_{ET}\left(T-T_{0}\right),\;K\left(T\right)=K_{0}-K_{0}k_{ET}\left(T-T_{0}\right)$$

where *k*_ET_ is the variation coefficient of the Young's modulus *E* over temperature. *E*_0_ and *K*_0_ are initial Young's modulus and spring stiffness, respectively. Then the natural resonant frequency of the silicon micro-machined gyroscope would be changed as follows:

(9) $$f\left(T\right)=\sqrt{K(T)/m}=\sqrt{K_{0}\left(1-k_{\text{ET}}\left(T-T_{0}\right)\right)/m}=f_{0}\sqrt{1-k_{\text{ET}}\left(T-T_{0}\right)}$$

where *f*_0_ is the resonant frequency of proof mass *m*. Meanwhile, the Q factor of any mode can be expressed as:

(10) $$Q=\frac{d}{\mu A}{\left(mK\right)}^{1G2},\mu =\frac{\sqrt{2}\pi d^{2}\mu_{0}pd}{k_{b}T}$$

where *d* is the fluid thickness, *A* is the plate area parallel with the substrate, *p* is the ambient pressure, *k_b_* is Boltzmann constant, *u*_0_ and *u* are original and changed viscosity coefficient of the gas respectively.

From above analysis, the natural resonant frequency and Q factor are both directly correlated with the sensitivity, stability, and other static and dynamic performances, so these factors would all be affected by temperature variation. Besides, the performance of the electronic components in drive and sense modes would also be influenced by temperature variation.

### 4.2. Establishment of the Compensation Model

From the above analysis, the complication of the influence on the zero bias of silicon micro-machined gyroscope is obvious, and it is different to deduce the precise math expressions theoretically. Therefore, it is relatively easier to derive the relationship expression between zero bias and temperature through lots of temperature experiments. The silicon micro-machined gyroscope was used in temperature experiment. The experimental steps are as follows: Firstly, the gyroscope is put into the temperature controlling casing, and the temperature is dropped down to -40 °C. Secondly, the zero bias of gyroscope is measured every 5 °C and sampled for one minute at each temperature point. Thirdly, the average zero bias value is calculated at each point, and the processed data is plotted in a graph. Lastly, the precise math expressions with least square fitting method is figured out from -40 °C to 80 °C. In order to reduce the fitting error and corresponding the polynomial orders, the fitting expressions of three orders term could be divided into three segments as follows:

When −40 °*C* ≤ *T <* −10 ° then:

(11) $$V_{\text{bias}}=0.4813+0.00719T-0.000735T^{2}+0.0000416T^{3}$$

When −10 °*C* ≤ *T < 50* °*C* then:

(12) $$V_{\text{bias}}=0.4313-0.00629T+0.00121T^{2}+0.00000642T^{3}$$

When 50 ° *C* ≤ *T <* 80 ° *C* then:

(13) $$V_{\text{bias}}=-673.07+42.879T-1.0146T^{2}+0.01053T^{3}$$

The polynomials in three temperature segments are stored in the memory of microprocessor, and in the compensation process, the microprocessor would calculate the compensation values according to the real-time temperature value measured by digital temperature sensor inside the gyroscope box, and at the same time the microprocessor calculates the desired signal by subtracting the compensation value from the sampled gyroscope signal.

### 4.3. The Design of Temperature Compensation System

**Figure 8.**
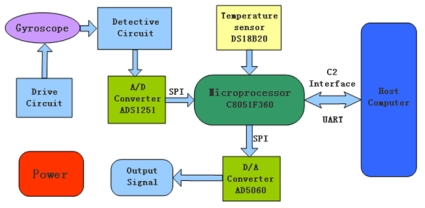
The hardware setup of the temperature compensation system.

Due to the accurate relationship between the zero bias and the ambient temperature of the gyroscope, the actual temperature compensation can be realized by designing a hardware platform. Figure 8 shows the block diagram of the main hardware modules: the detected uncompensated analog signal of the gyroscope is converted into digital signal by an ADS1251 AD converter (Texas Instruments Company). Meantime, the temperature value is measured by a DS18B20 digital temperature sensor (Dallas Semiconductor) and transmitted to a C8051F360 microprocessor (Silicon Labs Company) which is the core of whole system. The fitting polynomials are previously stored in the flash memory of the C8051F360 and used to calculate the compensation value in terms of the measured temperature, and then the compensated gyroscope signal is converted into analog signal by AD5060 (Analog Device Company) and transferred out. To facilitate debugging, the PC can communicate with the C8051F360 through UART and download successfully complied program into the C8051F360 by C2 interface. These components are all low power with high performance.

1. DS18B20 is a kind of 1-wire digital temperature sensor, with a wide measuring range from -55 °C to +125 °C, 9 - 12 bit digital temperature readout, and accuracy of ±0.5 °C. In the design, 12 bit resolution is chosen, and 3.3 V power supply is applied. Data acquision (DQ) pin is connected to pin P1.6 of the C8051F360.
2. Because the gyroscope signal is quite weak, a high-accuracy, wide dynamic range of 24-bit δ-Σ structure ADS1251 AD converter, which can achieve 19 bits of effective accuracy when its readout rate is up to 20 kHz, is ideal for this requirement. It is a single channel converter, with SO-8 seal, very small volume, and 2-wire serial interface, which can be directly connected to the microprocessor through a Serial Peripheral Interface (SPI).
3. According to the analog output requirement of the system, after converted by A/D and then compensated in the microprocessor, the compensated digital signal should be transmitted out again through a D/A converter. In this design, AD5060 with a 3-wire serial interface, which can communicate with microprocessor by SPI, is used to achieve outputting gyroscope angular rate signal compensated. C8051F360 has only one SPI port, which has to be connected with both ADS1251 and AD5060. Apart from SCLK, different data pins of SPI are connected with each separate pin SDATA for data transmission respectively, even at the same time. Though ADS1251 and AD5060 use the same SPI interface of the microprocessor, there is no conflict between them because an optimal program will enable them to work orderly based on time-division-multiplexing. In addition, a special pin of AD5060 will be connected to the microprocessor, which is used to generate an interrupt request to inform that the data conversion is over. The AD5060 can achieve 16 bits of effective accuracy for D/A conversion.
4. In order to facilitate the observation when debugging and recording data during the temperature experiments, the UART serial port is used to communicate with host computer in this design, port Tx and port Rx are configured respectively to the relevant pins of the microprocessor by using the overlapping switch function. The UC-5 debugging adapter (New Hualong Corporation) is connected to the debugging interface C2 of C8051F360 and an USB port connecter with the host computer is used to download the program to the internal flash memory of the C8051F360. A tiny-6 pin interface embedded in the printed circuit board (PCB) circuitry is connected to the standard 5 mm × 2 mm interface through a connector designed, which will realize not only downloading program conveniently in the debugging process, but also a reduction of area of the PCB and decreased volume in favor of miniaturization.

**Figure 9.**
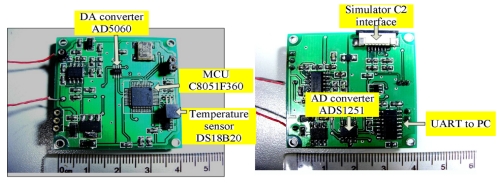
The PCB photos of the temperature compensation system.

A temperature compensation system using a C8051F360 microprocessor is thus designed. The gyroscope signal would be compensated by subtracting the compensation values which can be calculated through the expressions in terms of the temperature values measured. The PCB photos of the temperature compensation system are shown in Figure 9. In this paper, A/D and D/A are only used in digital compensation system. In Figure 5, the gyroscope's circuit in sense mode is pure analog using many discrete amplifiers which is independent of Figure 9. The compensated signal is processed by the circuit (see Figure 9) according to the detected temperature outside, and retransferred to analog signal with sum of gyroscope's output analog signal. In this implementation, Figure 9 shows only the circuit of digital temperature part while the packaged gyroscope is shown in later Figure 11.

## 5. Experimental Results

**Figure 10.**
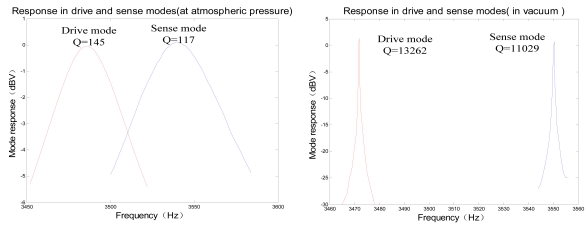
Response in drive and sense modes.

A SMG with the structure of changing-area capacitor detection is adopted in this paper, and the quality factor of a SMG (named as E17 sealed in metal can package working at an ambient pressure of 10 Pa) in two modes is above 10,000, so its sense mode detection essentially attains better linear performance and high sensitivity. Its responses in drive and sense modes (at atmospheric pressure and in vacuum) are shown in Figure 10. Single side driving and single side detection method is applied to make both vibrating amplitude and frequency highly stable in drive mode circuit. Meanwhile the dual-channel closed-loop detection method is utilized to achieve high precision detection in sense mode circuit. A miniature prototype scheme based on PCB technology has been realized with the dimention of 40 mm × 40 mm × 30 mm and power consumption less than 200 mW. The testing results demonstrate that the useful signal and quadrature signal would not interact with each other because of their phase decoupling. Under the condition of the scale factor of 9.6 mV/^°^/s, in full measurement range of ± 300deg/s, the zero bias stability attains 15 ^°^/h with worse-case nonlinearity of 400 ppm, the minimum measurable angular rate is 0.02^°^/s, the noise equivalent rate is 0.01^°^/s, and the noise is 0.0024^°^/s/√Hz by ALLAN variance analysis.

**Figure 11.**
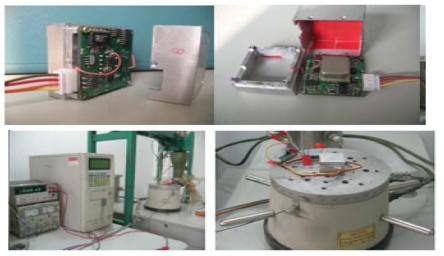
Miniature microgyro prototype.

**Figure 12.**
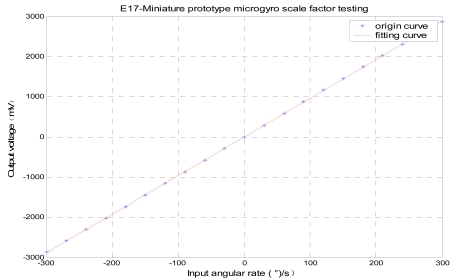
Scale factor testing.

**Figure 13.**
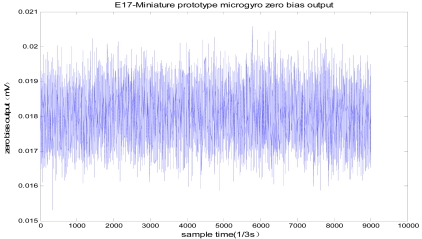
Zero bias stability testing.

**Figure 14.**
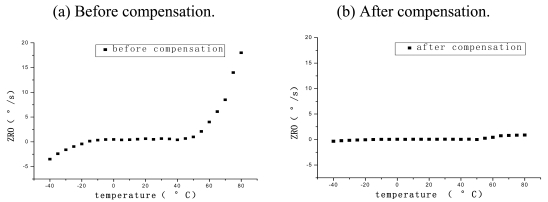
Efforts of temperature on ZRO before and after compensation.

A miniature prototype of SMG based on PCB and its testing platform are shown in Figure 11. Some important performances such as scale factor and zero bias stability are shown in Figures 12 and 13. The digital temperature compensation results are shown in Figure 14. The temperature experiment tests show that the influence of temperature variation on the zero bias of silicon micro-machined gyroscope is greatly eliminated and the zero bias is reduced to one tenth of the original after compensation from -40 °C to 80 °C. Table 2 generalizes the overall performance index of a miniature microgyroscope prototype at different temperature. In summary, the experiment results testify that the miniature prototype of SMG can be realizable with a satisfied performance.

## 6. Conclusions

A miniature vacuum-packaged SMG with symmetrical and decoupled structure was designed to prevent unintended coupling between drive and sense modes. To ensure high resonant stability and strong capacity of resisting disturbance, simplified self-oscillating closed-loop circuit include AGC loop and high frequency carrier modulation is implemented to reach a high precision in drive mode. The application occasion of the gyroscope is complicated, usually affected by many kinds of disturbances such as interference, over shock, vibration, temperature and so on. In addition, the bandwidth of SMG is approximately decided by the designed frequency difference between drive and sense modes. In order to improve the accuracy and reliability and broaden the system bandwidth, an improved dual-channel closed-loop detection method is adopted in this paper, which can ensure the stability and reliability of silicon micro-gyroscope's output by testing all kinds of specifications of SMG. Finally a miniature prototype of SMG with temperature compensation based on PCB is designed successfully. The experiment results testify that the miniature prototype SMG is realizable with good performance. Nevertheless, some important performances such as startup time of SMG and intelligent function of self detection and calibration will be perfected through further efforts.

## Figures and Tables

**Table 1.** Effect of the PID compensator's parameters on the system bandwidth.

*PID compensator parameters*	$\frac{0.002s^{2}+0.5s+1}{s}$	$\frac{0.0015s^{2}+2s+1}{s}$	$\frac{0.0025s^{2}+3s+1}{s}$	$\frac{0.005s^{2}+3s+2}{s}$
*K_p_*	0.5	2	3	3
*T_i_* (s)	0.5	2	0.3	1.5
*τ* (s)	0.004	0.0075	0.0025	0.0025
**Simulated bandwidth** (Hz)	60	99.8	146	205

**Table 2.** Performance index of miniature microgyro prototype.

**Technical data**		**Value**
**Performance(+25 ^°^C)**	*Scale factor*	9.6 mV/^°^/s
	*Bias stability*	15 ^°^/h
	*Noise*	0.0024^°^/s/√Hz
	*Noise equivalent rate*	0.01^°^/s
	*Dynamic range*	± 300^°^/s
	*The minimum measurable angular rate*	0.02^°^/s
	*Linearity*	≤ 400 ppm
	*Bandwidth*	> 80 Hz
**Power supply**	*Supply voltage*	± 5 V
	*Current dissipation*	20 mA
**Environment(-40 ^°^C∼ + 80 ^°^C)**	*Bias stability*	3^°^/s
	*Shock survival*	1000 g
	*Temperature drift*	< 0.03^°^/s/^°^C
